# A General Review of Methodologies Used in the Determination of Cholesterol (C_27_H_46_O) Levels in Foods

**DOI:** 10.3390/foods12244424

**Published:** 2023-12-10

**Authors:** Ashwell R. Ndhlala, Arzu Kavaz Yüksel, Neslihan Çelebi, Hülya Öztürk Doğan

**Affiliations:** 1 Green Biotechnologies Research Centre, School of Agricultural and Environmental Sciences, University of Limpopo, Private Bag X1106, Sovenga 0727, South Africa; ashwell.ndhlala@ul.ac.za; 2 Department of Food Technology, Technical Sciences Vocational School, Atatürk University, Erzurum 25030, Turkey; 3 Department of Chemical Technology, Vocational School of Technical Sciences, Ataturk University, Erzurum 25030, Turkey; nes25@atauni.edu.tr (N.Ç.); hdogan@atauni.edu.tr (H.Ö.D.)

**Keywords:** cholesterol, foods, determination methods, disease

## Abstract

Cholesterol (C_27_H_46_O) is a lipid-derived substance found in lipoproteins and cell membranes. It is also one of the main sources for the production of bile acids, vitamin D, and steroid hormones. Today, foods are evaluated by consumers not only according to their taste and nutritional content but also according to their effects on consumer health. For example, many consumers choose foods according to their cholesterol level. The cholesterol in the food can directly affect the blood cholesterol level when consumed, which can lead to cardiovascular diseases. High levels of cholesterol can lead to diet-related human diseases such as cardiac arrest, paralysis, type II diabetes, and cerebral hemorrhage. In societies with high living standards, interest in and consumption of foods that lower or have low cholesterol levels have increased recently. Accordingly, efforts to increase the variety of foods with reduced cholesterol levels are on the rise. This has indirectly led to the accurate measurement of cholesterol levels in blood and food being of great importance. Classical chemical, enzymatic, colorimetric, polarographic, chromatographic, and spectrophotometric methods; enzymatic, nonenzymatic, and electrochemical sensors; and biosensors are used for the determination of cholesterol in foods. The purpose of this review is to reveal and explore current and future trends in cholesterol detection methods in foods. This review will summarize the most appropriate and standard methods for measuring cholesterol in biological components and foods.

## 1. Introduction

**Figure 1:**
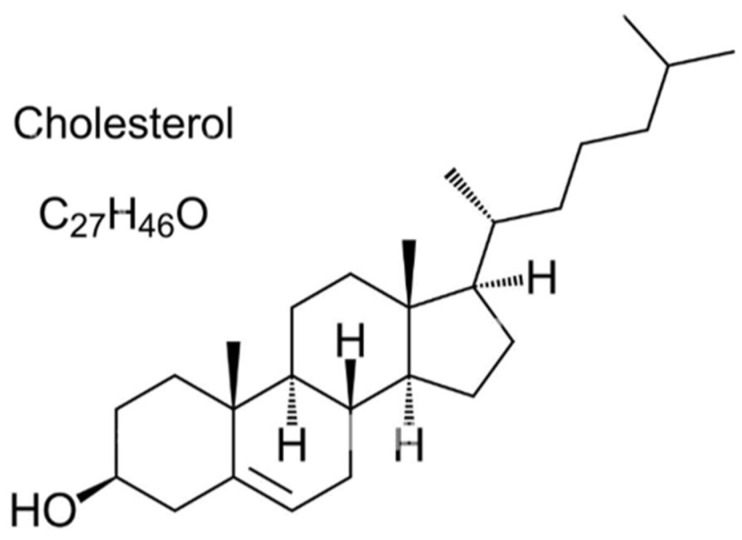
The general chemical structure of cholesterol.

Cholesterol (C_27_H_46_O) represents the main part of milk fat sterols. It is a mono-olefinic, secondary, high molecular, and complex alcohol [1]. Cholesterol is an alcohol-like steroid found in human and animal organisms’ cells and cell membranes, and is carried in the blood plasma. It is located in the structure of the cell wall and is known as one of the most essential sterols because it contributes to intercellular signal transmission (Figure 1). Cholesterol is of vital importance in the human metabolism. It was first discovered in gallstones, in 1754, hence its name, which was derived from the Greek words chole (bile) and stereos (solid) and the suffix -ol in chemistry [2].

Humans obtain cholesterol mainly from animal foods. However, a very small part of it in the body is obtained from foods; the rest of it is synthesized within the body. Although it is found in every cell of the body, its density is higher in organs and tissues that synthesize it, especially in the liver, spinal cord, brain, and arteries [3,4,5,6,7]. Its function is to modulate dietary fat absorption in the small intestine (as well as bile acids). It is one of the basic components of nerves and cell membranes. It is also a precursor for steroid hormones produced in the adrenal cortex, female and male sex hormones, and vitamin D, and is an essential substance for the development and growth of mammals [1]. Apart from animals, cholesterol is an essential compound also found in plant and fungal organisms. It plays an important role in the structure of cell membranes, blood lipoproteins, and the biosynthesis of steroid hormones, vitamins, and bile acids [8]. Its determination is very important not only for clinical examination but also for food quality control [9,10].

The presence of high levels of cholesterol in the blood results in its build up on the walls of blood vessels, causing the hardening and narrowing of the arteries, a medical condition called atherosclerosis. There are generally two types of cholesterol, defined by their carrier molecules. High levels of cholesterol carried by low-density lipoprotein (LDL), mostly referred to as the ‘bad cholesterol’, are the most harmful. The other type, carried by high-density lipoprotein (HDL), is called ‘good cholesterol’. HDL is commonly found in the brain synapses, playing a key role in the immune system and protection against cancer [4,11]. Higher cholesterol concentrations in the blood, especially the ones associated with LDL, cause some health problems including arteriosclerosis, hypertension, coronary heart, and kidney disease [12,13,14,15,16,17]. On the other hand, low concentrations of cholesterol can cause anemia and fatigue syndrome. In addition, cholesterol sometimes combines with bile pigments and leads to the complications with gallstones [6,18,19,20].

The normal level of cholesterol in a normal adult human body ranges from 150 to 200 mg/dL. Significant amounts of cholesterol are synthesized by the body per day to compensate for the loss occurring in excretion and other ways [21,22,23]. A daily intake of cholesterol of about 300 mg for adults is recommended by food safety authorities [24,25].

To control cholesterol levels in the body, it is necessary to pay attention to several factors [26,27,28]. These include but are not limited to food and nutrition as well as lifestyle such as habits that control blood pressure and body weight including smoking habits and increasing physical activity. This lowers plasma cholesterol concentrations and plasma lipids [29].

Dietary habits have a significant effect on the amount of cholesterol in the blood. The dietary intake of high-animal-fat foods instead of the plant-based intake of fruits, vegetables, and dairy products causes an increase in the level of cholesterol in the blood [30]. Although it has been stated that dietary fat intake has positive effects on human health, it is known to have a very significant effect on coronary heart disease [31]. Determining total cholesterol in serum is important for clinical measurements. When a high amount of cholesterol accumulates on the walls of various vessels and causes closure of the vessels, it results in the thyroid gland working less efficiently, causing thyroid disorder, diabetes, and jaundice [32]. The amount of cholesterol in different foods is given in Table 1.

The accurate measurement of cholesterol in the body is important in the diagnosis and treatment of cholesterol-based conditions. Modern amperometric and voltametric measurement techniques are increasingly used in the measurement of cholesterol. Basic research on biological materials and chemicals has been the driving force in the development and application of analytical technologies for the determination of cholesterol [33].

Measuring the presence of cholesterol in foods has been the subject of extensive research. In general, the production of electrodes for cholesterol detection is of great importance in terms of clinical tests. Additionally, monitoring the cholesterol level in blood and food is a critical parameter for diagnosing and preventing many diseases [34]. Researchers have thus far developed different methods to monitor cholesterol, including colorimetry [4,35]. These are chromatographic [36,37,38,39], fluorometric [39,40], and chemiluminescent methods [41]. In general, many of these mentioned methods require expensive instrumentation and complex preparation procedures to precipitate lipoproteins, or lack an acceptable sensitivity and selectivity.

Various colorimetric, polarographic, chromatographic, spectrophotometric, and biosensor methods are used in the determination of cholesterol. However, these are generally time-consuming and expensive systems. Measurements made via gas and liquid chromatography are the most suitable for cholesterol detection in terms of separating cholesterol from other similar compounds as well as determining its quantity [42]. Therefore, it is of great importance to develop systems that can determine the amount of cholesterol more accurately, in a shorter time, and at a lower cost. Generally, conventional methods such as colorimetry, spectrophotometry, fluorimetry, polarography, thin layer chromatography, gas chromatography, and high-performance liquid chromatography are used to measure cholesterol levels in the sample under investigation [43,44]. The majority of current sensors used are capable of adequately detecting free or esterified cholesterol. However, their originality is poor, complex, expensive, labor-intensive, and time-consuming [22]. In the last decade, enzymatic, non-enzymatic, and redox mediator-based sensors have been developed for cholesterol detection in enzymatic systems, enzymes such as cholesterol oxidase (ChOx) or cholesterol esterase (CE) catalyze the hydrolysis of cholesterol ester, resulting in the formation of fatty acids and free cholesterol. Because of the complexity of the matrix, chromatographic measurements with different types of detectors are the most common methods for cholesterol determination [45,46]. The main advantages of chromatographic methods are high selectivity, low LOD (depending on the detection used), and high accuracy. On the other hand, these techniques traditionally have disadvantages such as high cost and high personnel requirements [47,48]. 

Sensors that can only measure cations and anions with the classical electrochemistry system have enabled the determination of many substances with the inclusion of a biomaterial in the system. Biosensors find usage in applications such as bacteria and virus determination, agriculture, veterinary, biomedical sector, toxic gas analysis in mining enterprises, food production and analysis, drug analysis, military applications, process control, environmental protection and pollution control, clinical diagnosis, bioreactor control, agriculture veterinary, and industrial waste [49,50,51]. With the developing technology, biosensors, especially enzymatic biosensors, are used in hospitals and the food industry. Biosensors can be used for complex parameters such as the detection of foreign substances in foods, freshness, and aroma control [52,53,54]. In addition to this, biosensors can be used in the fight against drugs and preventing the misuse of drugs [55]. Although biosensors have high specificity and speed, they require the development of an enzyme stabilization method. This is one of the main disadvantages of this type of system. The instability of the enzyme poses a problem in obtaining accurate and precise results [56]. Proper orientation and high surface biocompatibility of the enzyme play an important role in facilitating electron transfer between the enzyme and the electrode surface. In these sensors, random orientation may lead to a decrease in the concentration of active enzymes on the electrode surface, which may result in a decrease in the sensitivity of the biosensor used [57]. In this regard, it is clear that non-enzymatic sensors that provide direct cholesterol signals have some advantages compared to biosensors, such as more advanced methodological features, simpler structure, lower cost, and longer shelf life.

This study aims to reveal the methods applied for the determination of cholesterol in food matrices and to reveal simple, fast, and sensitive methods by scanning different studies and using the data obtained in these studies.

## 2. Cholesterol Determination Methods in Foods

Recently, many new methods have been developed to detect the amount of cholesterol in various materials. Cholesterol can be detected by a variety of analytical methods, such as gravimetry, colorimetry, fluorimetry, chromatography, and enzymatic and non-enzymatic sensors. These methods can be examined in four main categories: (1) classic chemical tests, (2) colorimetric and fluorometric enzymatic tests frequently used in test kits and automatic plate readers, (3) analytical methods such as gas and liquid chromatography or mass spectrometry, and (4) enzymatic and non-enzymatic sensors. 

Classical chemical methods are relatively simple and inexpensive to apply compared to others but require multi-step procedures. Enzymatic assays involve the use of expensive enzymes, but their limits of detection (LODs) are generally low. Methods such as chromatography and mass spectrometry are the most accurate and sensitive methods. However, they require expensive equipment and long sample preparation preprocessing [58].

Details of these methods are explained comprehensively in the following subsections.

### 2.1. Detection of Cholesterol Using Enzymatic and Non-Enzymatic Methods

A wide variety of methods have been reported to monitor cholesterol in biological fluids and foods [59,60,61,62]. Enzymatic and non-enzymatic electrochemical determination methods to determine cholesterol have also been recently introduced [24]. 

Cholesterol was thought to be electrochemically inactive until the late 1980s [63]. In 2005, methods that first provided the direct oxidation of cholesterol and then indirect cholesterol oxidation methods (using an electron mediator) were developed [64,65]. 

Cholesterol oxidase, less commonly cholesterol esterase, and cholesterol modified with nanomaterials have been widely evaluated as sensing materials in electrochemical sensors [57]. Each of these approaches varies significantly, and their general use is quite limited because cholesterol oxidation products must be identified separately for each chosen electrolysis environment. Many new methods have been introduced for the characterization of direct oxidation products of cholesterol in non-aqueous media [63,65,66].

### 2.2. Determination of Cholesterol in Foods by Enzymatic Methods

This type of analysis relies on the presence of an enzymatic reaction to determine total cholesterol levels in food or other materials. The first enzymatic test capable of detecting cholesterol in serum was introduced in 1974 [67]. Enzymatic tests have been widely used since then, and more recently test kits and automated analyzers are now widely used [68]. In these methods, esterified cholesterol is first hydrolyzed to free cholesterol by cholesterol esterase. The resulting free cholesterol is then oxidized to cholesta-4-en-3-one by the cholesterol oxidase enzyme. Hydrogen peroxide is produced as a byproduct in this reaction and can be easily detected using high-sensitivity colorimetric or fluorometric probes [67,69,70].

### 2.3. Detection of Cholesterol in Foods by Chromatographic Methods

#### 2.3.1. Detection of Cholesterol in Foods with HPLC

The most common techniques for the nonenzymatic removal of cholesterol from foods are HPLC and other techniques such as GC-MS and LC-MS [38,71].

These mentioned methods are extremely sensitive and selective; however, they require complex sample preparation procedures and expensive equipment. The measurement of total cholesterol is generally made using chromatographic or enzymatic methods. The most commonly used method is gas chromatography, although some HPLC methods are also popular [72,73]. However, the determination of cholesterol by HPLC has received less attention than measurement by GC. The application of HPLC with UV detection to determine cholesterol has been limited in a complex sample environment, as the poor absorption of cholesterol at low wavelengths poses a problem in spectrophotometric detection [74]. It has been determined that gas chromatography (GC) is more sensitive, especially in determining cholesterol in food matrices [75,76,77,78,79,80]. These drawbacks encountered in HPLC measurements can be easily overcome by using high-performance liquid chromatography (HPLC) and especially reversed-phase HPLC. Additionally, the main advantage of HPLC over GC is that it is performed at low temperatures and prevents cholesterol oxidation [81]. 

Compared to HPLC, cholesterol determination by GC is more laborious. For example, it has disadvantages such as the derivatization of cholesterol compounds, the need to verify the reliability of measurement using internal standards before use, sample preparation being time-consuming (involving steps such as saponification and extraction, with a chromatographic run taking approximately 25 min), and being a costly method. In addition, the GC instrument is operated at a higher temperature than HPLC, which induces the formation of cholesterol oxides [38,81]. 

UHPLC and HPLC may be ideal analytical techniques for measuring cholesterol in food matrices because they are more sensitive, cost-effective, and less time-consuming than other traditional methods. 

The most common detector used for cholesterol detection in HPLC is the diode array detector (DAD). Studies have found that the most suitable HPLC detector to measure cholesterol levels in foods is a diode array detector (DAD), and other detectors such as ultraviolet (UV), fluorescence detection, evaporative light-scattering detection, infrared detection, and electrochemical detection have also been reported to be suitable for measurement [38,75,76,77,80,82,83]. Reverse-phase HPLC combined with a UV or DAD detector is the most common technique. In general, Analyst software 1.6.2 and Sciex OS 3.1 software drawing programs are used to prepare chromatograms [84]. In the literature, it has been reported that columns with a particle size of 5 microns can be used in most analytical methods, but it has been stated that the use of columns with a particle size of 3 or 4 microns may be appropriate [85].

The most convenient and often used method for sample preparation in liquid chromatography is the direct saponification of the sample followed by extraction of the unsaponifiable residue into a nonpolar solvent [86]. In this method, direct saponification is preferred because of the possibility of converting non-polar fatty acid esters to polar products by effectively removing them by multiple extractions with n-hexane [38,86,87]. Different options for extraction include single-stage extraction with toluene [82] or three-stage extraction with diethyl ether. It is also recommended to use a mixture of polar and non-polar solvents to enable a more efficient extraction of cholesterol from food matrices, in which cholesterol is often bound by many other biological compounds such as proteins, lipoproteins, and phospholipids [82]. 

Saponification and extraction processes are very important in determining the cholesterol ratio by HPLC. The saponification process is one of the most important steps in obtaining cholesterol purified from other components. In this process, potassium hydroxide is the most common solvent used to separate cholesterol from fatty acids [75,82,88]. The mixture is then washed with ultrapure water to remove these compounds, and the resulting cholesterol remains in the extracted solution layer for analysis [75]. Hexane is the most common reagent used for the extraction of cholesterol in complex food matrices such as egg yolks, due to its low polarity compared to toluene, which produces an emulsion.

#### 2.3.2. Detection of Cholesterol in Foods with GC-MS

Gas chromatography (GC) is also one of the most commonly used analytical techniques for the quantification of cholesterol and other sterols. However, although GC columns are a very effective method for the separation of cholesterol, sometimes problems may arise in the separation of cholesterol due to its similarities with other sterols [82,88]. 

It is widely accepted that this method is more reliable, sensitive, and accurate than other methods. In chromatographic columns, additional selectivity can be added to separate cholesterol from inhibitory sterols. Additionally, another advantage of this method is that a low volume of sample (tens of mL) is required for analysis [89].

The AOAC has published 994.10, an official method for the analysis of cholesterol in foods by GC-flame ionization detector after saponification and derivatization with trimethylchlorosilane [90]. In general, procedures applied in GC require the extraction of total lipids, separation of solvents, saponification of cholesterol esters, detailed solvent extraction of unsaponifiable material, repeated washing concentration of the analyte, and appropriate derivatization before GC analysis [90]. These process steps are quite burdensome in terms of both labor and materials and require increasingly expensive supply, recovery, and disposal costs. Although newer methods based on direct saponification of the sample have been developed and some steps have been eliminated, this method remains laborious and costly [91]. Although the lipid extraction and saponification steps are laborious, the derivatization reagents are unstable, and cholesterol is thermally decomposed in the GC column, the method is based on sound scientific principles. This method is still considered one of the most reliable methods.

#### 2.3.3. Electrospray Ionization Tandem Mass Spectrometer (ESI)

ESI is not an effective method for ionization to measure neutral free sterol molecules such as cholesterol. The molecular ions of the sterol molecules are easily fragmented in the [M + H]^+^ ion source. For that reason, they are very difficult to detect in the matrix using this method. In contrast, cholesterol ester species tend to form more stable ammonium adduct ions [M + NH_4_]^+^, which can be detected successfully [92].

#### 2.3.4. Matrix-Assisted Laser Desorption/Ionization Mass Spectrometry (MALDI)

MALDI is an effective ionization method for neutral free sterol molecules. Recent studies have provided increasing evidence that MALDI-MS can be used for cholesterol measurements. In a study conducted by Hidaka et al. [93], MALDI-TOF (time-of-flight) MS was used to analyze cholesterol in human serum lipoproteins. 

#### 2.3.5. Ambient Ionization Mass Spectrometer

In recent years, a method known as ambient ionization mass spectrometry (AIMS) has been developed for the chemical analysis of cholesterol in biological systems [94]. The ambient ionization technique operates at atmospheric pressure, in real time, and requires minimal sample pretreatment for rapid mass spectrometric analysis. Desorption electrospray ionization (DESI) can be used directly for real-time direct analysis (DART) and quantitative cholesterol measurements. It is also a technology that is twice as fast and more effective than direct ESI approaches for cholesterol ionization [95].

#### 2.3.6. Removal of Cholesterol from Foods by Nonenzymatic Methods

Cholesterol replacement therapy is required for the treatment of some inborn errors of metabolism. Therefore, most cholesterol determination techniques target blood and blood serum. The determination of cholesterol in foods is extremely important for health. Over the last decade, a wide variety of cholesterol sensors have been developed, including enzymatic, non-enzymatic, and redox mediator-based sensors. 

In enzymatic systems, the hydrolysis of cholesterol ester is catalyzed by using enzymes such as cholesterol oxidase (ChOx) or cholesterol esterase (CE), resulting in the formation of fatty acids and free cholesterol. Enzymatic sensors demonstrate high sensitivity and selectivity. However, the enzymes have a short lifespan and are easily denatured during immobilization. Additionally, their activities are easily affected by temperature, pH value, and toxic chemicals [96,97]. Non-enzymatic cholesterol sensors do not face the same limitations and problems as enzymatic ones. Electrode surfaces modified with metals, metal oxides, or composites have electrocatalytic functions. The most important feature of non-enzymatic electrodes is the use of nanomaterials with high surface-to-volume ratios that provide good interaction with external reagents, high conductivity, and excellent biocompatibility. Therefore, they are interesting components for bringing electrochemical devices to the nanoscale [98]. In this regard, it is very important to understand the latest developments in nanomaterial-based electrochemical cholesterol sensors and to follow future developments. The main feature of non-enzymatic electrodes is the use of high-surface-volume nanomaterials that interact well with external reagents and have high conductivity and good biocompatibility. Electrochemical- and nanomaterial- based sensors represent an important alternative to traditional methods in terms of their low cost, portability, high sensitivity, stability, and repeatability [99].

In recent years, electrochemical sensors have been accepted as a good alternative to traditional methods due to their low cost, reduced size, portability, high sensitivity, and shorter detection time [85,100]. Due to these properties, it may be very useful to develop an electrochemical sensor that can detect cholesterol in food and clinical samples. The most important consideration when developing a sensor system for any material is sensitivity. Various redox nanoparticles, including metallic nanoparticles and nanowires, magnetic nanoparticles, carbon nanomaterials, conductive polymers, and their hybrids, are used to increase sensitivity due to rapid electron transfer, which facilitates the conversion of an irreversible oxidation process into a reversible one [6,85,101,102,103,104,105].

### 2.4. Electrochemical Sensors

Electrochemical sensors are preferred because they are simple, are low-cost, have high accuracy, and have high and fast sensitivity levels. Carbon-based electrodes are widely used in electrochemical analysis because of their advantages such as the ease of surface modification and low background current [106,107]. 

The electron transfer rates of electrochemical sensors are generally lower than electrodes made of noble metals [108]. The electrochemical activity of carbon-based electrodes against some analytes can be increased by the anodic oxidation of the surfaces, resulting in the formation of new oxidized functional groups. In bare electrodes, a high voltage is required for cholesterol oxidation [63]. Overvoltage occurring in chemically modified electrodes (CMEs), popular in electro-analytical chemistry, can be significantly reduced using electrochemical sensors. The authors of [107,108,109,110] reported that they obtained a new sensor for rapid cholesterol determination via the electrochemical deposition of 1,4-diacetylglycoluryl (DPADGU) diphosphonic acid onto the carbon-containing electrode surface. The DPADGU used here catalyzes the oxidation of cholesterol. The electrochemical oxidation step is a complex process and is controlled by both diffusion and adsorption. However, the prepared sensor proved to be highly selective when tested in the presence of complex substances such as cholecalciferol, retinyl acetate, tocopherol acetate, albumin, lactose, uric acid, lactic acid, and ascorbic acid. Under optimum operating conditions (phosphate buffer pH 6.8), the developed sensor demonstrated a reliable linear DPV response from 1 to 200 μmol L^−1^, high sensitivity of 20 μAmolL^−1^ cm^−2^, low detection limit of 1.53 μM, and reasonable selectivity. This sensor, which has good performance, requires simple manufacturing, and is low-cost, is very promising for the food industry [108,109].

#### Nanomaterial-Based Electrochemical Sensors

Electrochemical sensors are a very attractive method for detecting glucose, cholesterol, cancer, and infectious diseases due to their high sensitivity, low cost, functionality, easy nanosize microfabrication, low energy requirements, and easy control. The development of nano biosensors is fraught with enormous challenges, such as ensuring longevity and native biocompatibility with the monitoring of analytes. Additionally, random signals resulting from nonspecific adsorption events can cause significant problems, especially in diagnostic analyses. In nanotechnological sensors, it is necessary to create and design structures with a size below 100 nm. Changes can be made to the optical, magnetic, and electrical properties; size; shape; and composition of nanostructures. These attributes can facilitate improvements in the biocompatibility, sensitivity, and specific binding of biomaterials [108,109].

Electrochemical biosensors (EBS) have recently received great attention for the management of cholesterol (CHO) in healthcare. Quantitative analysis of cholesterol is performed using amperometric data of the linear calibration chart. Determining the cholesterol in the blood is very important to control the health problems that may occur when it reaches abnormal levels. Electrochemical sensors have been recognized as a highly selective method for cholesterol detection due to their simplicity, low cost, wide working range, and high sensitivity. These sensors are analytical devices that combine the specificity of biomolecules with an electronics-based physicochemical transducer to convert a biochemical signal into a measurable electrical signal. A conventional EBS contains three electrodes, a saturated Ag/AgCl electrode, a platinum wire, and a glassy carbon electrode or metal plate electrode, which serve as a reference and counter electrode, respectively. In achieving EBSs, cholesterol oxidase is widely used as a biosensing material immobilized by a different type of electroactive material. Metal nanoparticles were effectively immobilized with cholesterol oxidase due to their high conductivity properties with different binders and were used in the surface modification of working electrodes to detect cholesterol. Metal nanoparticles have been reported to be used in various combinations with conductive polymers, graphene, chitosan, and graphene oxide–carbon nanotubes with high electrical conductivity, effective surface area, and rapidity to improve EBS performance [111].

The effectiveness and efficiency of EBSs generally depend on the modification of electrode surfaces working with different electroactive coating materials. The morphology of the produced working electrode is characterized by scanning electron microscopy, transmission electron microscopy, atomic force microscopy, X-ray diffraction, and energy-dispersive X-ray spectroscopy. The performance of the produced electrodes for determining the cholesterol detection amount is investigated by square wave voltammetry and differential pulse voltammetry methods [108,109,111].

Functional nanomaterials have attracted significant attention in various research fields (especially in the healthcare system) due to their easy control, high chemical and environmental stability, biocompatibility, and unique optoelectronic and sensing properties. The recognition properties of nanomaterials can be used to identify biomolecules such as cholesterol. In the last decade, significant advances have been made in the production of cholesterol biosensors, the main component of which are nanomaterials [57,99].

### 2.5. Possibilities of Using Biosensors in Measuring Cholesterol in Foods

Among the various existing methods used to detect cholesterol, biosensors are a relatively simpler, faster, more sensitive, and more specific method [61,112]. Biosensors are analytical devices that consist of a transducer and a biological element. Bioelements such as enzymes, antibodies, nucleic acids, receptors, organelles, and microorganisms interact with the analyte under study, and the concentration of substances or other biological response parameters are converted into an electrical signal [113].

In biosensor production, the immobilization of enzymes on electrodes is important. The high performance of the amperometric biosensor simply immobilizes the enzyme on the electrode and stimulates electron transfer in sensor fabrication using mediators, promoters, or other special materials. The majority of cholesterol biosensors have been developed based on the electrochemical reduction of hydrogen peroxide (H_2_O_2_) due to their simplicity and specificity [12].

Biosensors can be used in the fabrication of biosensing devices due to their biocompatibility, high viscosity, non-toxicity, and solubility in acidic aqueous environments. Redox-active enzymes are often preferred to test the electron activity and catalytic abilities of cholesterol [114]. Electrochemical biosensors fabricated based on immobilized enzymes have found wide applications for the detection of large masses of biological substrates. Newer systems do not use intermediaries but directly monitor the redox behavior of the enzyme at the electrode surface. This behavior produces or consumes electrons and can increase signal conduction at the working electrode. Gholivand and Khodadadian immobilized ChOx and catalase (CAT) on a graphene/ionic liquid-modified glassy carbon electrode (GR-IL/GCE) [115].

In a study conducted by Ferri et al. [116], the behavior of a multi-enzymatic electrodic system for the detection of glucose and choline based on horseradish peroxidase (HRP) was investigated. The electrodic system captured by horseradish peroxidase quickly detected the presence of these analytes, which are involved in the reactions in which hydrogen peroxide is produced, even without the need for additional mediators [117].

Enzymatic sensors exhibit high sensitivity and selectivity. On the other hand, enzymes have a short lifespan and are easily denatured during immobilization. Moreover, their activities are affected by temperature, pH value, and toxic chemicals [96,97].

#### Metal Nanoparticle (MNP)-Based Cholesterol Biosensors

MNP has very good electronic properties, and for this reason, its use in the production of various biosensors has been investigated [118]. Electrodes coated with up to 0.5 nM Pt/Graphene and Pt/CNT have been developed for amperometric measurement used to detect cholesterol. It has been reported that cholesterol can be detected as specific molecules down to the nano level by digitalonin gold nanoparticles [119]. These results allowed us to elucidate various aspects of redox, pointing to a process involving the biosystems under study and the potential of these polymers for the construction of next-generation biosensors. The excellent sensitivity of cholesterol was obtained from the biosensor based on the enzyme-immobilized microtubular ZnO/ZnS heterostructure [120].

The electrochemical photoelectrochemical cholesterol biosensor based on graphene-embedded titanium nanowires has also been reported to have an outstanding sensitivity of 6 μM [121]. Cholesterol biosensors with graphene oxide and Pd nanostructure have been successfully used for the analysis of total cholesterol in human serum and milk fat [122]. Wang et al. [123] used a nanocomposite consisting of molybdenum disulfide nanoparticles for the ultra-measurement of cholesterol. Current developments in electrochemical biosensors are characterized by an application-oriented approach, and studies have led to the formation of miniaturized biosensors by transforming electrode surfaces.

## 3. Conclusions

This review gives detailed information about the methods used to determine cholesterol in foods. In addition, the strengths and weaknesses of the mentioned methods are revealed. Comparisons were also made about the sensitivity levels of the classical chemical, enzymatic, colorimetric, chromatographic, and spectrophotometric methods, and also enzymatic, nonenzymatic, and electrochemical sensors, and biosensors.

## Figures and Tables

**Table 1:** Cholesterol amounts in different foods.

Foods	Cholesterol (mg/100 g)
Brain	2353
Egg yolk	1260
Kidney	803
Egg	396
Liver	360
Butter	240
Cheese	160
Cream	109
Veal	100
Chicken meat	98
Beef	60
Breast milk	25
Cow’s milk	12.3
Yogurt	12.2
Skim milk	3

## Data Availability

Not applicable.
